# Knee Replacement Bandaging Study (KReBS) evaluating the effect of a two-layer compression bandage system on knee function following total knee arthroplasty: study protocol for a randomised controlled trial

**DOI:** 10.1186/s13063-019-3344-1

**Published:** 2019-05-08

**Authors:** Liz Cook, Matthew J. Northgraves, Caroline Fairhurst, Sarah Ronaldson, David J. Torgerson, Jonathan Kent, Mike Reed

**Affiliations:** 1 York Trials Unit, Department of Health Sciences, University of York, Heslington, York, YO10 5DD UK; 2 Northumbria Healthcare NHS Foundation Trust and University of York, North Tyneside General Hospital, Rake Lane, North Shields, Tyne and Wear NE29 8NH UK

**Keywords:** Knee replacement, Arthroplasty, Compression bandage, Randomised controlled trial

## Abstract

**Background:**

Data from a feasibility study suggest that the use of an inelastic, short-stretch compression bandage following total knee arthroplasty is a safe technique that may improve patient-reported health outcomes, and that it is feasible to recruit to a full-scale study.

**Methods:**

We will conduct a randomised controlled trial (RCT) of 2600 adult patients, which has 80% power to detect a 1 point difference in the Oxford Knee Score (a patient self-reported assessment of knee pain and function) at 52 weeks. Short stretch compression bandaging will be compared with standard wool and crepe bandaging following total knee arthroplasty. Recruitment will take place in orthopaedic units across the United Kingdom. Secondary outcomes include the EuroQol 5 Dimensions (EQ-5D)-5 L and EQ-5D-3 L scores, pain, length of hospital stay, and complications.

**Discussion:**

The Knee Replacement Bandaging Study (KReBS) is a large study which aims to contribute to the evidence base for informing clinical decisions for the use of compression bandaging following knee arthroplasty.

**Trial registration:**

International Standard Randomised Controlled Trial Register, ISRCTN 87127065. Registered on 20 February 2017.

**Electronic supplementary material:**

The online version of this article (10.1186/s13063-019-3344-1) contains supplementary material, which is available to authorized users.

## Background

Total knee arthroplasty (TKA) is a common and highly successful operation in the management of osteoarthritis [1]. Between 2003 and 2016 more than 870,000 entries for primary TKA were submitted to the 14th National Joint Registry report for England, Wales, Northern Ireland, and the Isle of Man [2]. Post-operative knee swelling is common and results in decreased functional performance which in turn can lead to delayed rehabilitation, an increase in length of hospital stay, and a decrease in patient satisfaction [3].

Compression bandage therapy is an established treatment of venous ulcers and lymphoedema, but efficacy in TKA remains unclear due to conflicting results in the literature and heterogeneous methodology. A feasibility study has been performed [4, 5] and data from that study suggested that the use of an inelastic, short-stretch compression bandage following TKA is a safe technique and that it was feasible to enlist surgical teams to recruit suitable patients. A large randomised controlled trial (RCT) is needed to establish effectiveness.

## Methods/design

This is a pragmatic, multicentre RCT investigating the use of a two-layer compression bandage system compared with a standard care (wool and crepe) bandage following TKA.

The study includes an economic evaluation.

### Objective

The objective is to determine the effectiveness and cost-effectiveness of a two-layer compression bandage compared with a standard wool and crepe bandage applied post-operatively on patient-reported outcomes in TKA patients.

### Setting

The Chief Investigator (CI) obtained initial agreements to participate from orthopaedic units in National Health Service (NHS) Hospital Trusts in the United Kingdom across a range of urban and rural areas with the provision of adding further interested centres. The pragmatic design of the trial and wide clinician involvement ensures immediate applicability and generalisability of the trial findings. Two thousand and six hundred patients will be recruited from approximately 26 secondary care orthopaedic units from across the UK. Table 1 shows a list of all participating hospital trusts sites that have been set up to recruit patients into the trial.

**Table 1:** KReBS participating hospital trust sites

	Study sites
1.	Northumbria Healthcare NHS Trust
2.	Gateshead Health NHS Foundation Trust
3.	York Teaching Hospital NHS Foundation Trust
4.	North Cumbria University Hospitals NHS Foundation Trust
5.	The Dudley Group NHS Foundation Trust
6.	County Durham and Darlington NHS Foundation Trust
7.	Royal Devon and Exeter NHS Foundation Trust
8.	Hampshire Hospitals NHS Foundation Trust
9.	North Tees and Hartlepool NHS Foundation Trust
10.	East and North Hertfordshire NHS Trust
11.	East Kent Hospitals University NHS Foundation Trust
12.	South Tees Hospitals NHS Foundation Trust
13.	Maidstone and Tunbridge Wells NHS Trust
14.	Norfolk and Norwich University Hospitals NHS Foundation Trust
15.	Taunton and Somerset NHS Foundation Trust
16.	Manchester University NHS Foundation Trust
17	Warrington and Halton Hospitals NHS Foundation Trust
18.	Wrightington, Wigan and Leigh NHS Foundation Trust
19.	Western Sussex Hospitals NHS Foundation Trust
20.	City Hospitals Sunderland NHS Foundation Trust
21.	Dorset County Hospital NHS Foundation Trust
22.	Betsi Cadwalder University Health Board
23.	Golden Jubilee National Hospital
24.	Stockport NHS Foundation Trust
25.	University Hospitals Coventry and Warwickshire NHS Trust
26.	Epsom and St. Helier University Hospitals NHS Trust

### Study participants

Patients are eligible to participate in this study if they: 1) are scheduled for primary total knee arthroplasty; 2) present at a participating trial site; 3) are aged 18 years and over; and 4) are willing and able to provide written informed consent.

Patients will be excluded from this study if they: 1) are unable to provide informed consent; 2) have a history of peripheral vascular disease; 3) have a history of peripheral neuropathy; 4) have a history of, or current, venous ulceration; 5) have absent foot pulses; 6) are planned same-day discharge joint replacement patients; 7) are scheduled for revision knee arthroplasty; 8) are scheduled for Unicondylar or patellofemoral joint knee arthroplasty; 9) are prescribed regular concomitant high-dose anti-coagulant medication (patients on routine thromboprophylaxis can be included); 10) are unwilling to provide informed consent; 11) lack mental capacity and are therefore unlikely to comply with data collection; 12) are scheduled for bilateral knee replacement; or 13) have been previously recruited into KReBS (now scheduled for knee replacement on the opposite leg).

Additional clarification to accompany exclusion 9 was issued to participating sites as follows:

- This exclusion applies to high-risk patients, most likely with metal heart valves who require anticoagulation through the peri-operative period. These patients will usually be given intravenous heparin instead of their regular medications and should be clarified with the operating surgeon if unsure. In usual practice these will be rare.
- The vast majority of patients on anti-coagulation will be those taking warfarin, apixaban, rivaroxaban, etc. for deep vein thrombosis (DVT), atrial fibrillation (AF), or stroke prevention. They will have stopped this prior to the operation and will restart it following the operation. These patients should be included in the study.

### Trial interventions

#### Standard care (control) group

The bandage will be applied over the routine surgical wound dressing.

The control treatment is one layer of soft synthetic bandage applied from the proximal tibia to distal femur covered by a further layer of crepe bandage prior to or after tourniquet deflation, with 50% overlap of each layer. Cryotherapy can be applied over this if part of routine care. The bandage should be removed between 24 and 48 h post-operatively with the dressing remaining in situ.

#### Intervention group

The bandage will be applied over the routine surgical wound dressing. Intervention bandage is applied from the toes upwards. The application of the bandage from thigh to groin requires removal of the tourniquet first, keeping the leg elevated until the bandaging is complete.

Initially a foam inner bandage (Coban 2, 3 M UK) is applied from the toe to the groin on the affected leg with minimal overlap. The second layer of compression bandage (Coban 2, 3 M UK) is applied at 50% stretch and with a 50% overlap of bandage to ensure adequate compression in the application.

To ensure homogeneity in bandage application, the operating surgeons will be shown a training video on correct application. The compression bandage is removed on the planned day of discharge, at least 24 h following application. For patients not already discharged, the compression bandage will be removed at 48 (± 4) hours post-operation.

The bandages can be removed before 24 h if the patient finds them very uncomfortable, or in the event of clinical need or any adverse occurrence that would require their removal.

#### Rehabilitation

For both the intervention and standard care groups, patients will receive physiotherapy as per routine care.

### Outcome measures

Table 2 outlines the time points at which the outcomes are assessed. These outcomes are described below.

**Table 2:** Study assessment schedule

	Study period						
	Enrolment	Allocation	Post-allocation				
Timepoint	Baseline (pre-randomisation)	Randomisation	Day of surgery	Approximately 10 days post-surgery	Week 4	Month 6	Month 12
Enrolment							
Eligibility screen	x						
Informed consent	x						
Allocation		x					
Interventions							
Compression Bandage			x				
Standard care Bandage			x				
Assessments							
Body mass index	x^a^						
Demographic data	x^a^						
Comorbidities	x^a^						
Oxford Knee Score	x^a^					x^a^	x
EQ-5D-3 L	x^a^					x^a^	x
EQ-5D-5 L	x						x
Pain scores (via SMS)				x	x		x
Basic health economics data (i.e. healthcare resource use)							x

*EQ-5D* EuroQol 5 Dimensions

^a^From routine data where possible

### Primary outcome

The primary outcome and end-point for the trial is the Oxford Knee Score (OKS) total score at 12 months from randomisation. OKS is a patient-reported outcome measure specifically designed and developed to assess function and pain after TKA surgery.

### Secondary outcomes

- OKS at 6 months post-surgery.
- EuroQol 5 Dimensions (EQ-5D)-3 L index score at 6 months post-surgery.
- EQ-5D-3 L index score at 12 months post-randomisation.
- EQ-5D-5 L index score at 12 months post-randomisation.
- Pain scores at 10 days, 4 weeks, and 12 months post-surgery (collected via SMS message for those participants that opt into receiving SMS messages as part of the study).
- Length of hospital stay.
- Proportion of each patient group that: has to return to theatre within 30 days of surgery for any reason; is readmitted to hospital within 30 days of surgery for any reason; suffers a pulmonary embolism (PE) requiring inpatient hospitalisation within 30 days of surgery; suffers a DVT requiring inpatient hospitalisation within 30 days of surgery.

### Participant timeline

**Fig. 1:**
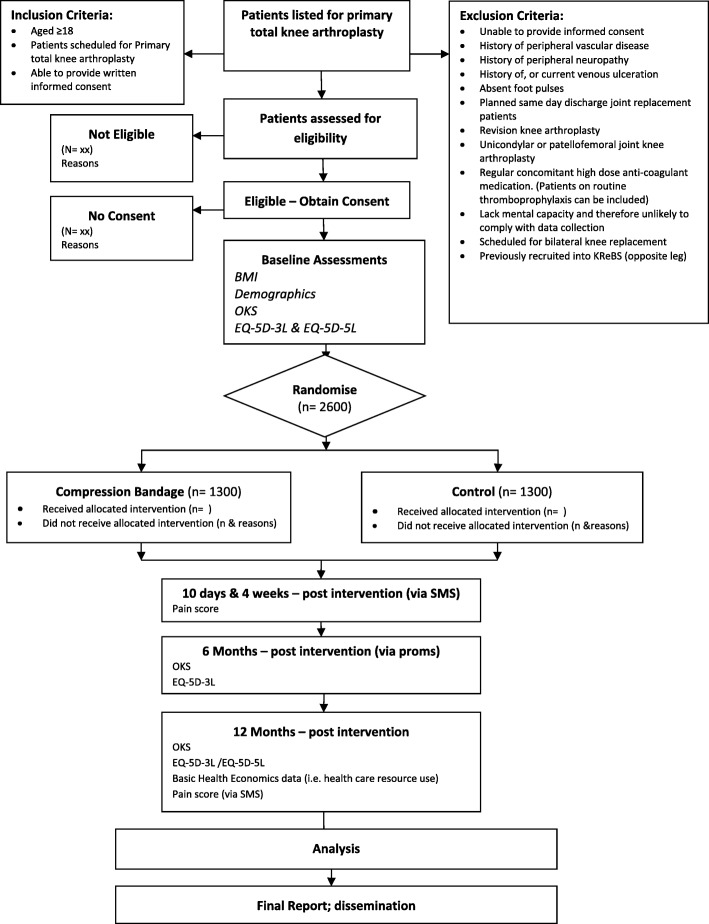
KReBS study flow chart. Figure illustrating the process of enrolling participants into the study, the interventions being compared, and timing of assessments for the participants in the trial. BMI body mass index, EQ-5D EuroQol 5 Dimensions, OKS Oxford Knee Score, PROM Patient-Reported Outcome Measure

Figure 1 illustrates the process of enrolling participants into the study, the interventions being compared, and timing of assessments for the participants in the trial.

### Sub-studies

#### Text message

An embedded RCT will be undertaken to evaluate the effectiveness of a personalised text message prompt, including the participant’s name, compared with a standard text message on postal response rates to the 12-month questionnaire. This is separate to the collection of pain scores via SMS (all patients that consent to receiving text messages will be asked to return pain scores).

Text messages have been found to be effective for improving completion and return of questionnaires in trials [6]. Little research exists on the use of personalised text messaging for improving trial response rates.

Participants will be randomised in a 1:1 ratio to receive a standard text message, or to personalised text messages which includes their name with their 12-month follow-up questionnaire. Generation of the allocation sequence will be undertaken independently by a researcher not involved with the delivery of the text messages, and will use simple randomisation. Participants are able to opt out of receiving text messages without affecting participation in the main trial. Therefore, the sample size of the text sub-study will be constrained by the number of participants that consent to receiving text messages.

Participants will be sent the text messages at the same time as they are expected to receive their postal follow-up questionnaire (i.e. 2 to 4 days after the questionnaire is sent). Text messages are likely to be sent using secure UK-based text message gateway software such as that provided by Intelli Software (https://www.intellisoftware.co.uk/).

The primary outcome of this embedded trial will be the proportion of participants who return their questionnaire. Secondary outcomes will be time to response and completeness of response.

#### Pen sub-study

We will undertake an embedded RCT to evaluate the effectiveness of including a pen with the 12-month questionnaire on response rates. Participants allocated to the intervention group will receive a pen with the University of York logo on it, whilst control participants will receive no pen. Participants will be randomised using simple randomisation in a 1:1 ratio.

All participants in the KReBS main trial will be eligible to be included in the pen sub-study. There are no exclusion criteria.

The primary outcome will be the proportion of participants who return the 12-month questionnaire. Secondary outcomes will be time to response and completeness of response.

#### EQ-5D-3 L and -5 L order

We will undertake an embedded RCT to evaluate whether the order in which the EQ-5D-3 L and -5 L appear in the 12-month questionnaire impacts on the participant responses. Participants will be allocated using simple randomisation in a 1:1 ratio to either receive a questionnaire in which the EQ-5D-3 L appears before the -5 L, or a version in which the EQ-5D-5 L appears first. All participants due to be sent their 12-month questionnaire will be included in this embedded trial.

#### Northumbria blood loss sub-study

Blood loss during and following TKA reduces a patient’s haemoglobin and haematocrit counts whilst increasing the need for blood transfusion. It has been hypothesised that the use of a compression bandage will act as a tamponade and help reduce this loss [7]. Acute post-operative pain is also a concern and, from previous studies, compression bandaging may help decrease a patient’s post-operative pain [8].

A subset of main trial participants undergoing TKA in Northumbria NHS Foundation Trust will have their routinely taken pre- and post-operative full blood counts and transfusion data reviewed as well as the first 24 h post-operative pain scores and breakthrough analgesia requirements.

The primary outcome measure of the embedded blood loss sub-study will be the difference in haemoglobin level changes between the two arms. Secondary outcome measures will be the difference in haematocrit level changes and inpatient transfusion rates. Secondary endpoints, additional to those specified in the main trial protocol, will be length of stay, 24-h pain scores, and breakthrough analgesia requirement as well as readmission and complications rates.

Our initial sample size calculations were for 207 patients to observe a difference of 1 g of haemoglobin (90% power, SD 2.1). However, we adjusted this calculation to take into account the pre- and post-test correlation and a potential clustering around the surgeon.

A sample size of 156 (78 in each group) will give us 90% power to detect a difference in post-operative haemoglobin level (the primary outcome measure) between the intervention and control groups of 0.35 g/dl (SD 0.7, which equates to an effect size of 0.5), assuming a pre-post correlation of 0.7, 10% loss to follow-up, an average of 60 procedures per consultant, and an ICC of 0.01.

This sub-study is being undertaken as part of a student project and the results are to be reported separately to those of the main trial.

### Sample size

A standard deviation for the OKS of 8, an attrition rate of 15%, and a clinically important difference of 3 points were determined from a preliminary feasibility study. However, because the intervention is very inexpensive and low-risk it could be argued that a smaller difference than 3 may still be meaningful. Assuming a more conservative attrition rate (20%) than was observed in the feasibility trial and powering at 80% to detect a 1-point difference we need to recruit and randomise 2515 participants. We have decided to round this to 2600. Furthermore, the power of the study does not include the influence of covariates (e.g. baseline test score), which will correlate with the outcome scores and provide increased power from the adjusted regression analyses.

### Recruitment

All patients scheduled for TKA who are deemed eligible by their surgical team will be mailed information or given information on the trial at a pre-operative visit. Patients will be given sufficient time to accept or decline involvement. Patients will be given the opportunity to discuss the trial with research staff or their treating surgical team prior to their surgery. Following confirmation of eligibility, written informed consent will be obtained by research staff prior to surgery. Participants will be free to withdraw from the study at any time without affecting their routine care.

Strategies for achieving adequate participant enrolment to reach the target sample size include seeking advice from our patient representatives, sharing best practice with our Research Nurses, and regular discussion with our Principal Investigators (PIs).

Site staff will be provided with training at the site initiation visits to ensure adherence to the delivery of the interventions in the trial. During the trial, training and reminders will be implemented using e-mail bulletins, and discussion with the PIs and with Research Nurses. In addition, the Trial Co-ordinators will provide support and guidance to staff when required (e.g. when new staff join or replace existing site staff) and will seek clinical guidance from the Chief Investigator (CI) when necessary.

### Randomisation

Simple, equal randomisation without stratification or blocking will be used to generate the treatment allocation schedule. As the sample size of the trial exceeds 200 there is no loss of statistical efficiency compared with using more complex (restricted) randomisation methods [9].

When patients have given consent and their baseline forms have been completed, the Research Nurse or recruiting clinician will randomise them using the York Trials Unit’s (YTU) secure, web-based randomisation service. Patients will be allocated 1:1 to receive either compression bandaging or standard care (wool and crepe) bandaging, therefore ensuring allocation concealment and immediate unbiased allocation. Patients will be informed of their allocations as will the clinician managing each patient.

### Data management

Paper case report forms (CRFs) will be used to record all the information required from the protocol with the following exceptions: demographic, comorbidity, and complications data will be downloaded from Patient Administration Systems (PAS) at each site; pain scores will be collected from participants via SMS message where they have agreed to be contacted via this means; and Patient-Reported Outcome Measures (PROMs) at baseline and 6 months post-surgery (OKS and EQ-5D-3 L) will be obtained via the NHS Digital’s PROMS project (https://www.england.nhs.uk/statistics/statistical-work-areas/proms/) and reported via participating sites to YTU.

All data will be completely anonymised for the purposes of analysis and any subsequent reports or publications. For the purposes of ongoing data management, once randomised, individual participants will only be identified by trial identification numbers to maintain confidentiality.

All paper records will be kept in locked locations. All consent forms will be secured safely in a separate compartment of a locked cabinet. Essential trial documentation (i.e. the documents which individually and collectively permit evaluation of the conduct of a clinical trial and the quality of the data produced) will be kept with the Trial Master File and Investigator Site Files. At the end of the study, paper copies of data will be securely archived by participating sites and the University of York for a minimum of 5 years. Electronic data will be stored indefinitely.

Once YTU has completed the analysis and published all intended scientific journals, data will be made available for other researchers for secondary analysis upon request. Requests for access to data will be reviewed by the CI and study sponsor.

### Statistical analysis

A detailed analysis plan will be agreed with the combined Trial Steering Committee (TSC) and Data Monitoring and Ethics Committee (DMEC) at an early stage of the study, before all of the data have been collected. Any subsequent amendments will be clearly stated and justified. Analyses will be conducted following the principles of intention-to-treat (ITT) with outcomes analysed according to the patients’ original, randomised group irrespective of deviations based on non-compliance, and significance tests will be two-sided at the 5% level, unless otherwise stated.

The primary analysis will compare total OKS scores between the treatment groups at 12 months using a covariance pattern mixed model incorporating outcome data at 6 and 12 months, adjusting as fixed effects for age, gender, baseline score, time point, treatment group, and a treatment group-by-time point interaction, and hospital site as a random effect. Treatment effects in the form of an adjusted mean difference will be presented with an associated 95% confidence interval (CI) and *p* value for both time points (6 and 12 months). The primary end-point will be the treatment effect estimate at 12 months, while the treatment effect at 6 months will serve as a secondary outcome. Pain score (at 10 days, 4 weeks, and 12 months) will be analysed similarly.

The proportion of participants who, within 30 days of surgery, 1) return to theatre, 2) are readmitted to hospital, 3) experience a PE requiring hospitalisation, and 4) experience a DVT requiring hospitalisation will be compared between the two groups using mixed effects logistic regression models, adjusting for age and gender as fixed effects, and hospital site as a random effect. The treatment effect in the form of an odds ratio will be presented with an associated 95% CI and *p* value.

Length of hospital stay in days will be analysed via a mixed effect Poisson, or negative binomial, regression model as appropriate, adjusting for age and gender as fixed effects, and hospital site as a random effect. An incidence rate ratio, 95% CI and *p* value will be provided.

### Interim analysis

There are no planned interim analyses for the trial or stopping guidelines.

### Cost-effectiveness analysis

An economic analysis will be undertaken to determine the cost-effectiveness of compression bandages versus standard bandages following TKA. This will take the form of a cost-utility analysis, thereby incorporating the impact on patients’ health-related quality of life. The analysis will be conducted from the perspective of the UK NHS and Personal Social Services (PSS), in line with NICE recommendations.

Health benefits for the economic evaluation will be measured in terms of quality-adjusted life years (QALYs). The EuroQol EQ-5D-3 L will be used to obtain utility values for use in the cost-utility analysis. QALYs will be generated for each patient based on the area under the curve approach (Richardson G). EQ-5D data will be collected at baseline (pre-operatively) and 6 months post-operatively (both collected routinely via the National PROMS programme) and at 12 months (via a 12-month follow-up participant questionnaire). At baseline and 12 months, in addition to the EQ-5D-3 L, we will also collect EQ-5D-5 L data to enable a mapping exercise and to utilise the updated -5 L version of the EQ-5D. An analysis of the order in which the EQ-5D-3 L and EQ-5D-5 L appear in the 12-month follow-up questionnaire will be undertaken to assess ordering effects associated with the presentation of the two EQ-5D versions.

Costs will be assessed for the two groups; healthcare resource use data will be collected. Specifically, primary care resource use (i.e. General Practitioner (GP), nurse, physiotherapist, occupational therapist visits) and resource use in the hospital setting (i.e. hospital outpatient appointments, inpatient stays, day cases, and accident and emergency admissions) will be obtained using patient self-reported data from the 12-month follow-up questionnaire. Data obtained via routine data collection methods (e.g. outpatient visits) may also feed into the economic analysis if available. Unit costs will then be applied to the quantities of resources utilised, for example using NHS Reference Costs (Department of Health) and Unit Costs of Health and Social Care [10]. In addition, product/intervention costs such as bandages/equipment will be costed and information relating to complications will be incorporated for the two groups. Information on patients’ return to work/activities will also be collected.

The within-trial analysis will use regression methods to generate estimates of mean costs and health outcomes (i.e. QALYs), allowing for correlation between cost and outcome data, and will adjust for covariates. The results will be presented in terms of incremental cost-effectiveness ratios (ICERs), specifically the incremental cost per QALY. Future costs and outcomes will not be discounted due to the trial follow-up not exceeding 12 months. Multiple imputation methods will be used to handle missing data where needed [11]. Cost-effectiveness acceptability curves will be produced to explore the probability that compression bandages will be cost-effective at different cost-effectiveness thresholds [12]. Uncertainty will also be explored via sensitivity analysis to investigate the impact of underlying assumptions of the analysis and key cost drivers in terms of the cost-effectiveness results.

### Adverse event management

Adverse events related to the participant’s trial-related compression bandaging up to 1 month after their knee operation will be collected. Adverse events considered to be a consequence of the surgery will not be collected or reported in the context of this study. Symptoms relating to possible complications of surgery, inter-current illness, or any inpatient episode within 30 days of surgery will be collected. Other than for fatalities, this procedure does not apply to any other adverse events which may occur during the trial which are unrelated to the trial procedures.

The severity and likely relationship of any adverse events will be documented by the designated site clinician. An event is defined as ‘related’ if the event was due to the administration of any research procedure, whereas an ‘unexpected event’ is defined as a type of event not listed in the protocol as an expected occurrence. The relatedness of an event will be reviewed by the Chief Investigator and the Trial Steering Committee. Serious adverse events that are confirmed to be related to the research and are unexpected will be reported to the Research Ethics Committee (REC). All adverse events will be routinely reported to the Trial Management Group (TMG) and sponsor. The combined TSC/DMEC will be responsible for reviewing related and unexpected serious adverse events.

### Quality control

Northumbria Healthcare NHS Foundation Trust has agreed to be the lead sponsor for this project and take overall responsibility for the quality of study conduct. This study will be fully compliant with the Research Governance Framework and MRC Good Clinical Practice Guidance.

A rigorous programme of quality control will be undertaken. The day-to-day management of the trial will be the responsibility of the Trial Co-ordinator based at York Trials Unit. Regular meetings with the Trial Management Group will be held and will monitor adherence to the trial protocols at the trial sites.

Due to the low-risk nature of this study, approval will be sought from the funders to set up one Independent Steering and Monitoring Committee to undertake the roles traditionally undertaken separately by the TSC and the DMEC. This committee will comprise of an Independent Chair who will be a surgeon with expertise in knee replacement surgery, a statistician, a member of the Patient Group, the Chief Investigator, and Trial Coordinator/Manager. Other study collaborators may also attend the meeting. The independent members of the committee will be allowed to see unblinded data. The role of this committee will include the review of all serious adverse events which are thought to be treatment-related and unexpected. The committee will meet at least annually or more frequently if the committee requests.

## Discussion

### Protocol modifications

Important protocol modifications are those that are likely to affect, to a significant degree: the safety, physical or mental integrity of the subjects of the study; the scientific value of the study; or the conduct or management of the study. These substantial amendments will be submitted to the Health Research Authority (HRA) and REC for approval, having first been agreed with the Sponsor and TMG. Minor modifications to the protocol will be agreed with the TMG before submission for approval to the HRA. All amendments will be implemented in the NHS organisations in compliance with HRA guidance. Trial participants will be written to, if necessary, to explain any changes. All amendments, whether substantial or not, will be listed in the published Final Report.

### Dissemination

This protocol is being made publicly available. The results will be disseminated in international, open-access peer-reviewed journals, through the local networks and at national and international meetings in surgical care. A dissemination and publication policy will be developed with an agreement between partners including ownership and exploitation of intellectual property, and publication rights.

Following publication of the main trial findings, data will be made available for other researchers for secondary analysis upon request. Requests for access to data will be reviewed by the CI and study sponsor.

### Trial status

The current REC approved version of the protocol is version 3.0 (19 March 2018). This manuscript is a restructured and edited version of the current REC approved protocol to comply with the SPIRIT guideline. A completed SPRIT checklist can be found in Additional file 1. Recruitment into the KReBS trial commenced in March 2017 and is ongoing at the time of manuscript submission. To date, 2150 patients have been randomised (July 2018). Recruitment was originally scheduled to end in February 2018. Early indications were that recruitment targets would not be met due to early delays with the R&D approval process at several of the originally planned sites and lag time taken to recruit the first patient at several participating sites. An extension to recruitment was discussed and recommended by the TMG and TSC/DMEC and was agreed upon by the funders. The recruitment phase has been extended to the end of August 2018.

## Additional file

Additional file 1: SPIRIT 2013 checklist: recommended items to address in a clinical trial protocol and related documents. (DOC 120 kb)

## Abbreviations

CI: Chief Investigator
CI: Confidence interval
DMEC: Data Monitoring and Ethics Committee
DVT: Deep vein thrombosis
EQ-5D: EuroQol 5 Dimensions
ITT: Intention-to-treat
NHS: National Health Service
OKS: Oxford Knee Score
PAS: Patient Administration System
PE: Pulmonary embolism
PROMS: Patient-Reported Outcome Measures
PSS: Personal Social Services
QALY: Quality-adjusted life year
RCT: Randomised controlled trial
REC: Research Ethics Committee
TKA: Total knee arthroplasty
TMG: Trial Management Group
TSC: Trial Steering Committee
